# Diazoxide Improves Mitochondrial Connexin 43 Expression in a Mouse Model of Doxorubicin-Induced Cardiotoxicity

**DOI:** 10.3390/ijms19030757

**Published:** 2018-03-07

**Authors:** Michela Pecoraro, Michele Ciccarelli, Antonella Fiordelisi, Guido Iaccarino, Aldo Pinto, Ada Popolo

**Affiliations:** 1 Department of Pharmacy, University of Salerno, Via Giovanni Paolo II, 84084 Fisciano, Italy; mipecoraro@unisa.it (M.P.); pintoal@unisa.it (A.P.); 2 Department of Medicine and Surgery, University of Salerno, 84084 Baronissi, Italy; mciccarelli@unisa.it (M.C.); giaccarino@unisa.it (G.I.); 3 Department of Advanced Biomedical Sciences, Federico II University, 80138 Naples, Italy; antonella.fiordelisi@gmail.com

**Keywords:** diazoxide, doxorubicin, connexin 43, calcium homeostasis

## Abstract

Doxorubicin (DOXO) administration induces alterations in Connexin 43 (Cx43) expression and localization, thus, inducing alterations in chemical and electrical signal transmission between cardiomyocytes and in intracellular calcium homeostasis even evident after a single administration. This study was designed to evaluate if Diazoxide (DZX), a specific opener of mitochondrial K_ATP_ channels widely used for its cardioprotective effects, can fight DOXO-induced cardiotoxicity in a short-time mouse model. DZX (20 mg/kg i.p.) was administered 30 min before DOXO (10 mg/kg i.p.) in C57BL/6j female mice for 1–3 or seven days once every other day. A recovery of cardiac parameters, evaluated by Echocardiography, were observed in DZX+DOXO co-treated mice. Western blot analysis performed on heart lysates showed an increase in sarco/endoplasmic reticulum Ca^2+^-ATPase (SERCAII) and a reduction in phospholamban (PLB) amounts in DZX+DOXO co-treated mice. A contemporary recovery of intracellular Ca^2+^-signal, detected spectrofluorometrically by means of FURA-2AM, was observed in these mice. Cx43 expression and localization, analyzed by Western blot and confirmed by immunofluorescence analysis, showed that DZX co-treatement increases Cx43 amount both on sarcoplasmic membrane and on mitochondria. In conclusion, our data demonstrate that, in a short-time mouse model of DOXO-induced cardiotoxicity, DZX exerts its cardioprotective effects also by enhancing the amount Cx43.

## 1. Introduction

Anthracyclines are a class of antitumor drugs widely used for the treatment of a variety of cancers, such as breast cancer, lymphoma and melanoma [1]. Unfortunately, its usage is limited by the cumulative, dose-dependent cardiomyopathy. In fact, one major side effect of this class of chemotherapeutic drugs is cardiotoxicity [2], leading to dilated cardiomyopathy and heart failure [3]. The mechanism of DOXO-induced cardiotoxicity is multifactorial and it is associated with the production of ROS and oxidation of lipids, DNA and proteins [4], mitochondrial dysfunction, myofibril degeneration [5,6] and altered calcium handling by sarcoplasmic reticulum [7]. Until recently, only the long-term effects of Doxorubicin (DOXO)-administration had been studied. In fact, it was believed that DOXO-induced cardiotoxicity was only related to accumulation of the repetitive doses required by the treatments, so total DOXO concentration cannot exceed 500 mg/m^2^ [8]. Recent evidence, instead, indicates that the damage caused by anthracyclines on cardiomyocytes is an early event, already evident after a single administration [9,10].

In our previous study conducted in a short-time mouse model, we demonstrated that DOXO is able to induce a significant dysfunction in Ca^2+^ homeostasis and affects connexin 43 (Cx43) expression and localization [11].

Cx43 is a member of the connexin family, so it is involved in Gap Junction formation allowing cell–cell communication, but it is also expressed on mitochondrial membranes [12] where it forms hemichannels [13]. The physiological role of mitochondrial Cx43 (mitoCx43) has not been elucidated, but recent studies show that it modulates K+ influx to the mitochondrial matrix, mitochondrial respiration and reactive oxygen species (ROS) generation [13,14]. Indeed, mitoCx43 may play a role in mediating the cardioprotective effect of ischemic preconditioning. Protection by mitoCx43 has been linked to ROS generation, mitochondrial KATP channels, protein kinase C (PKC) signaling, and stimulation of translocase of outer membrane-20 (Tom20) that facilitates Cx43 transport [15], from cytosol to mitochondria with a mechanism that involves heat shock protein 90 (Hsp90) [16]. It is well known that mitochondria are the major effectors of cardioprotection by mechanisms that open the mitochondrial KATP channels, including pharmacological preconditioning by Diazoxide (DZX), a specific opener of mitochondrial K_ATP_ channels [17]. K_ATP_ channels are ubiquitously expressed. At physiological conditions these channels are inhibited by ATP that binds the Kir6 pore-forming subunit. During times of stress K_ATP_ channels are open, thus providing a “unique electrical transducer of the metabolic state of the cell” [18,19]. Studies conducted in multiple animal models as well as in human myocytes showed that pharmacologic opening of K_ATP_ channels is able to induce cardioprotection and mimics ischemic preconditioning [20]. In view of the specific role played by both DZX and Cx43 on mitochondria, this study aimed to investigate if the pretreatment with DZX could attenuate DOXO-induced cardiotoxicity and affects Cx43 expression and localization in a short-time mouse model.

## 2. Results

### 2.1. Cardiac Functions

Echocardiography was performed on mice at baseline and before sacrifice in order to analyze main cardiac parameters. As summarized in Table 1, DOXO administration affects Ejection Fraction (EF), Fractional Shortening (FS), Left Ventricular End-Diastolic-Diameter (LVEDD), and Left Ventricular End-Systolic Diameter (LVESD). In fact, DOXO-treated mice showed a reduction of FS and EF, exhibiting a decrease of cardiac systolic function, and an increase of LVESD and LVEDD compared to control mice. In DOXO+DZX co-treated mice a rise of cardiac functions was observed.

### 2.2. Diazoxide Administration Alters Calcium Homeostasis

**Figure 1:**
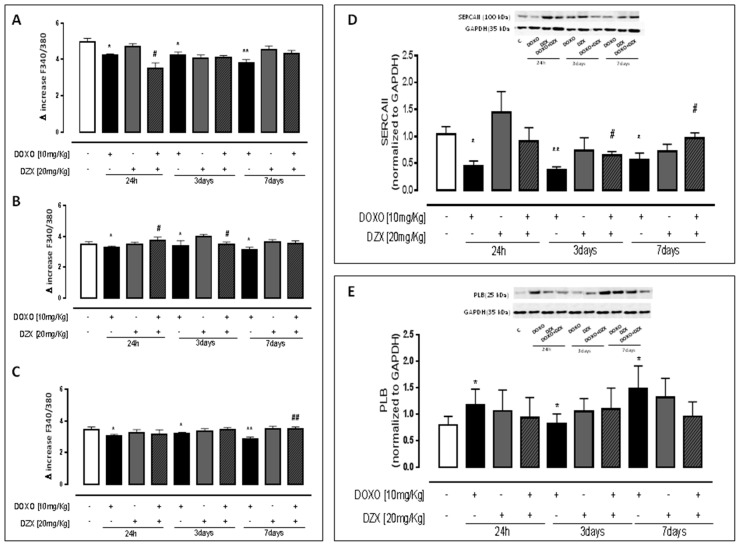
Effect of DOXO (10 mg/kg; i.p.) or DZX (20 mg/kg; i.p.) or combined DZX+DOXO treatment on calcium homeostasis. Mice received a single administration (1th group), two administrations (2nd group), or three administrations (3rd group) of DOXO (10 mg/kg; i.p.) or DZX (20 mg/kg; i.p.) or combined DZX+DOXO treatment and primary cardiomyocytes were isolated by enzymatic digestion. Intracellular calcium content in cell suspensions was evaluated by using ionomycin (1 μM) (**A**); reticulum calcium content was evaluated by means of thapsygargin (100 nM) (**B**) and mitochondrial calcium content was evaluated by using FCCP (50 nM) (**C**). Results were expressed as mean ± S.E.M. of delta (δ) increase of FURA-2 AM ratio fluorescence (340/380 nm) from at least three independent experiments each performed in duplicate. Data were analyzed by Student’s *t*-test. * *p* < 0.05, and ** *p* < 0.005 vs. control; # *p* < 0.05 and ## *p* < 0.005 DZX+DOXO vs. DOXO. Effect of DOXO or DZX or combined DZX+DOXO treatment on SERCA II (**D**) and PLB (**E**) expression. Mice received a single administration (1th group), two administrations (2nd group), or three administrations (3rd group) of DOXO (10 mg/kg; i.p.) or DZX (20 mg/kg; i.p.) or combined DZX+DOXO treatment and SERCA II and PLB amount was detected by Western blot analysis into tissue homogenates from mice; GAPDH amount was used as loading control. Values were expressed as mean ± S.E.M. from at least three independent experiments each performed in duplicate. Data were analyzed by Student’s *t*-test. * *p* < 0.05 and ** *p* < 0.005 vs. control; # *p* < 0.05 DZX+DOXO vs. DOXO.

Primary cardiomyocytes isolated from hearts of mice treated as previously described and from hearts of control mice were loaded with FURA2-AM in Ca^2+^-free incubation buffer to evaluate intracellular Ca^2+^ signal. Our data demonstrated that DZX-pretreatment significantly affected Ca^2+^ homeostasis. In fact, as indicated in Figure 1A, DOXO administration induced alterations in Ca^2+^ homeostasis by increasing intracellular free Ca^2+^ levels. Furthermore, as reported in Figure 1B,C, delta increase induced by both reticular ionophore (thapsigargin) and mitochondrial depletor (FCCP) in cardiomyocytes of DOXO-treated mice were significantly (*p* < 0.05) lower than those of control mice, indicating higher basal levels of Ca^2+^. On the contrary, in primary cardiomyocytes of DZX+DOXO co-treated mice, both reticular and mitochondrial Ca^2+^ signals were comparable to those of control mice.

SERCAII (Smooth Endoplasmic Reticulum Calcium ATP-Ase) protein is a Ca-dependent ATPasic pump that regulates the turn-over of Ca^2+^ ions in the intracellular environment, between the sarcoplasmic reticulum and the cytosol, and its activation involves the reuptake of Ca^2+^ in the sarcoplasmic reticulum. The activity of SERCAII is, in turn, regulated by the PLB polymeric protein, which inhibits SERCA II depending on the degree of phosphorylation of the individual monomers of PLB [21]. Western blot analysis performed on heart homogenates showed, as reported in Figure 1D, that in the hearts of DOXO-treated mice, that the SERCAII amount is reduced compared to the hearts of control mice. DZX administration promotes the expression of SERCAII. In fact, the amount of this protein in DZX-treated mice was comparable to those of control mice. The effect of DZX was evident even in DZX+DOXO co-treated mice, since the amount of SERCA in the heart of these mice was significantly (*p* < 0.05) higher than those of mice treated with DOXO alone. The amount of PLB in the heart of DOXO-treated mice was significantly (*p* < 0.05) higher than control mice. In contrast, in DZX+DOXO co-treated mice a progressive reduction of PLB amount was observed (Figure 1E).

### 2.3. Diazoxide Administration Affects Cx43 and pCx43 Expression and Localization

**Figure 2:**
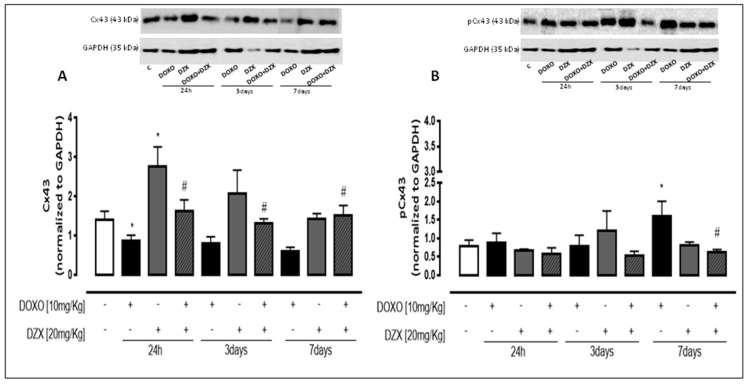
Effect of DOXO or DZX or combined DZX+DOXO treatment on Cx43 (**A**) and pCx43 (**B**) amount. Mice received a single administration (1th group), two administrations (2nd group), or three administrations (3rd group) of DOXO (10 mg/kg; i.p.) or DZX (20 mg/kg; i.p.) or combined DZX+DOXO treatment and Cx43 and pCx43 amount was detected by Western blot analysis of tissue homogenates from mice; GAPDH amount was used as loading control. Values were expressed as mean ± S.E.M. from at least three independent experiments each performed in duplicate. Data were analyzed by Student’s *t*-test. * *p* < 0.05 vs. control; # *p* < 0.05 DZX+DOXO vs. DOXO.

As reported in Figure 2A, Western blot analysis performed on heart lysates showed that DOXO-treatment induces a significant (*p* < 0.05) decrease of total Cx43 amount, also evident in mice that received a single DOXO administration. On the contrary, DZX significantly affected Cx43 expression. In fact, in the heart of DZX+DOXO co-treated mice the amount of Cx43 was significantly (*p* < 0.05) higher than those of mice treated with DOXO alone.

Connexin phosphorylation plays an important role in regulating biological function [22]. Western blot analysis showed a significant (*p* < 0.05) increase in the amount of Cx43 phosphorylated on Ser368 in DOXO-treated mice (Figure 2B) after seven days of treatment. DZX+DOXO co-treatment define a significant (*p* < 0.001) reduction of pCx43 compared to DOXO-treated mice. In fact, no significant differences are observed between DZX+DOXO co-treated mice and control mice. 

### 2.4. Diazoxide Administration Affects the Mitochondrial Amount of Cx43 and pCx43

**Figure 3:**
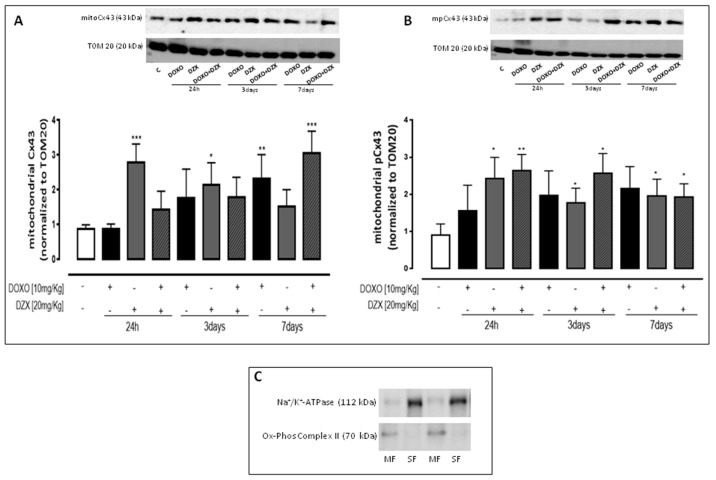
Effect of DOXO or DZX or combined DZX+DOXO treatment on mitochondrial amount of Cx43 (**A**) and mitoCx43 phosphorylated (**B**). Mice received a single administration (1th group), two administrations (2nd group), or three administrations (3rd group) of DOXO (10 mg/kg; i.p.) or DZX (20 mg/kg; i.p.) or combined DZX+DOXO treatment and amounts of mitoCx43 and mitoCx43 phosphorylated (mpCx43) were detected by Western blot analysis into tissue homogenates from mice; TOM20 protein amount was used as loading control. Values were expressed as mean ± S.E.M. from at least three independent experiments each performed in duplicate. Data were analyzed by Student’s *t*-test. * *p* <0.05, ** *p* < 0.005 and *** *p* < 0.001 vs. control. Representative Western blots of Na^+^/K^+^ ATPase and Ox-Phos Complex II were used as markers of sarcolemma (SF) and mitochondria (MF), respectively, to demonstrate the purity of the mitochondrial extracts (**C**).

Western blot analysis performed on mitochondrial lysates showed that DOXO-administration induces a significant (*p* < 0.05) increase of mitoCx43 amount at all experimental times. In the hearts of DZX+DOXO co-treated mice a further significant (*p* < 0.05) increase of mitoCx43 amount was observed (Figure 3A).

Regarding the amount of mitoCx43 phosphorylated on Ser 368 (Figure 3B) we observed an increase in the heart of DOXO-treated mice. It is important to note that in the heart of DZX+DOXO co-treated mice, the amount of mitoCx43 phosphorylated was significantly higher than those of DOXO-treated mice, but a trend to reduction was observed through time.

**Figure 4:**
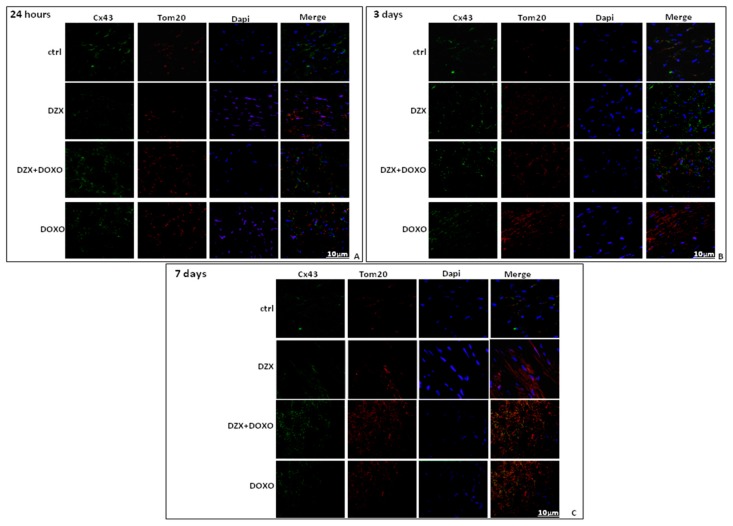
Effect of DOXO or DZX or combined DZX+DOXO treatment on Cx43 localization. C57BL/6j mice received a single administration (1st group), two administrations (2nd group), or three administrations (3rd group). Frozen myocardial tissue sections were stained with Anti-Cx43 (green), TOM20 (red) and nucleus with DAPI (blue) and were determined by Immunofluorescence assay at confocal microscopy for mitoCx43 localization. Scale bar 10 µm.

Immunofluorescence analysis performed on heart sections double-stained for Cx43 and TOM20, as a marker of mitochondria, confirmed an increased mitochondrial localization of Cx43 in the hearts of DZX+DOXO co-treated mice (Figure 4).

## 3. Discussion

DOXO is one of the most widely used and successful antitumor drugs, but its cumulative and dose-dependent cardiotoxicity limits its clinical application [23]. Mechanisms of DOXO-induced cardiotoxicity are very complex and remain elusive. They include a rearrangement of GJs, responsible for the cell-to-cell communication, leading to mitochondrial injury and promoting myocardial cell apoptosis [24,25], with an alteration of calcium homeostasis [11,26]. Mitochondrial functions in cardiomyocytes are preserved by action of mito K_ATP_-channels. In fact, DZX, an opener of mito K_ATP_-channels, is widely used for its cardioprotective effects [27]. 

This study aimed to investigate if opening of K_ATP_-channels by DZX is protective against DOXO-induced cardiotoxicity, in a short-term model of DOXO-induced cardiomyopathy in mice.

In our previous study, we have shown that even a short-term model administration of DOXO is able to induce significant changes in calcium homeostasis and alterations in Cx43 expression and localization [11]. So, we hypothesized that a cardioprotective drug given together with DOXO could mitigate these effects.

Echocardiography confirmed that DZX administration is able to restore major cardiac parameters such as Ejection Fraction (EF), Fractional Shortening (FS), Left Ventricular End-Diastolic-Diameter (LVEDD), and Left Ventricular End-Systolic Diameter (LVESD). In the cardiac ventricle, Cx43 regulates intercellular coupling, conduction velocity, and anisotropy [28] and is also responsible for action potential propagation [26]. In the heart of DZX+DOXO co-treated mice we observed a significant increase of Cx43 expression which may be responsible for the improvement of the observed cardiac functions. The heart rhythm is also guaranteed by right regulation of Ca^2+^ signaling. Here, we report that DOXO administration induces a significant dysfunction in Ca^2+^ homeostasis and, in agreement with data previously reported [29,30], we showed that DZX, by stimulating mitochondrial potassium flux, triggers cardioprotection by recovering mitochondrial and cytosolic Ca^2+^ accumulation and reducing the levels of free Ca^2+^ in cardiomyocytes. Intracellular Ca^2+^ concentrations are finely regulated by sarcoplasmic reticulum and mitochondrial coupling. Interactions between the sarcoplasmic reticulum and mitochondria are major determinants of efficient Ca^2+^ buffering and, thus, have an important role in cardiomyocyte physiology and dysfunction [31].

The improvement of reticular Ca^2+^ storage following DZX-pretreatment could be explained by the restoration of SERCA II and PLB levels that are dysregulated in DOXO-treated mice. SERCA II and PLB are involved in Ca^2+^ cycling, a critical process referring to the mobilization of intracellular Ca^2+^ in excitation-contraction coupling [32]. Defects in the regulation of these Ca^2+^-handling proteins are reported in hypertrophy, in heart failure [33] and also in DOXO-induced cardiomyopathy [34]. The antihypertrophic effects of DZX have been proven [35] and even if no studies report data on the direct correlation between DZX and SERCA II, we can speculate that SERCA II expression increases because DZX reduces the hypertrophy of cardiomyocytes. Mitochondrial Ca^2+^ is enhanced by mitoCx43 which is also overexpressed in the hearts of DOXO-treated mice, but which is even more expressed in the hearts of DZX+DOXO co-treated mice. 

The increase of mitoCx43 in cardiomyocytes can be induced by various stimuli, such as cellular stress and ischemic preconditioning, but its functional relevance is still unclear. It has been postulated that mitoCx43 is part of a multiprotein complex that somehow controls mitochondrial homeostasis and that forms hemichannels that serve as a conduit for ion flux [13], like Ca^2+^. Many authors indicate that mitoCx43 exerts cardioprotective effects by increasing mitochondrial calcium uptake and storage capacity that helps to delay a rise in cytosolic calcium levels [36].

So, our hypothesis is that the mitoCx43 overexpression observed in DOXO-treated mice is a defense mechanism put in place by cardiomyocytes to reduce cytosolic Ca^2+^ overload and the consequent cell death. In fact, under high intracellular Ca^2+^ conditions, mitochondria may function as a buffer to control cytosolic Ca^2+^ concentrations, thus, delaying cell death. DZX co-treatment enhances this self-defense mechanism by increasing mitoCx43 expression. The effect of DZX on mitoCx43 up-regulation has also been reported by Hou and co-workers [37] who demonstrated a strict correlation between mitoCx43 and mitoK_ATP_ channels. The important role played by mitoK_ATP_ on Cx43 expression has been also demonstrated in a rat model of acute myocardial infarction [38]. 

MitoCx43 can be phosphorylated on Ser368 by PKC and it has been shown that phosphorylation of Cx43 on Ser368 modulates the conductivity of the GJs [39], blocking the chemical coupling. This mechanism is considered a further “defense” against the propagation of injurious stimuli since phosphorylation of Cx43 by PKC induces the closure of hemichannel [37]. Here, in agreement with other studies, we have shown that DZX increases the phosphorylation on Ser368 of Cx43 by inducing PKC-ε translocation from the cytosol to mitochondria [27]. The DZX-induced PKC-ε activation is implied in several cardioprotective properties [40]. In human cardiomyocytes, PKC-ε is suspected to stabilize mitochondria, interacting with several targets [41], such as Cx43 [42,43]. On the basis of our data, we hypothesized that, in our experimental model, DZX pretreatment could form complexes with mitoCx43 and promote its phosphorylation at Ser368 [42,43], thus, inducing the closure of hemichannels. 

In conclusion, our data demonstrate that DZX represents a promising protective intervention to reduce the damage induced by DOXO. The cardioprotective effects of DZX are well documented. Here we reported that Cx43 could also be involved in these effects. In fact, the increase of Cx43 could be responsible for the improvement of cardiac functions observed in DZX pre-treated mice, while the increased expression of mitoCx43 and of mitoCx43 phosphorylated on Sr368 could be involved in the reduction of intracellular free-Ca^2+^ levels.

In conclusion, we can hypothesize that DZX exerts its cardioprotective effects by enhancing Cx43 expression and mitoCx43, thus, improving cardiac function and Ca^2+^ homeostasis altered in DOXO-induced cardiotoxicity.

## 4. Materials and Methods

### 4.1. Materials

DOXO was obtained from Baxter manufacturing S.p.a. (Officina di Sesto Fiorentino, Florence, Italy). DZX was obtained from Sigma (Milan, Italy) and all antibodies used were purchased from Santa Cruz Biotechnology (DBA, Milan, Italy).

### 4.2. Animals

Adult female C57BL/6j weighting 20–22 g, were purchased from Charles River (Lecco, Italy). All experimental protocols were approved by the Italian and European Community Council for Animal Care (DL. No. 26/2014 protocol number of Ministerial approval DGSAF 13788-A, 7 February 2015) and in accordance with the guidelines of the Guide for the Care and Use of Laboratory Animals of the National Institutes of Health.

### 4.3. Experimental Protocols

C57BL/6j mice were randomly divided in three groups (*n* = 12 for each experimental group). The doses used were 10 mg/kg [11] for DOXO and 20mg/kg for DZX [44].

First Group (24 h):

- 3 mice received a single DOXO administration (10 mg/kg i.p.) and were sacrificed 24 h after the treatment.
- 3 mice received a single DZX administration (20 mg/kg i.p.) and were sacrificed 24 h after the treatment.
- 3 mice were pretreated with DZX (20 mg/kg i.p.) and then received a single DOXO (10 mg/kg i.p) administration and were sacrificed 24 h after the treatment.

Second Group (3 days):

- 3 mice received DOXO (10 mg/kg i.p.) every other day and were sacrificed 3 days after the treatment.
- 3 mice received DZX (20 mg/kg i.p.) every other day and were sacrificed 3 days after the treatment.
- 3 mice were pretreated with DZX (20 mg/kg i.p.) and then received DOXO (10 mg/kg i.p.) every other day and were sacrificed 3 days after the treatment.

Third Group (7 days):

- 3 mice received DOXO (10 mg/kg i.p.) every other day and were sacrificed 7 days after the treatment.
- 3 mice received DZX (20 mg/kg i.p.) every other day and were sacrificed 7 days after the treatment.
- 3 mice were pretreated with DZX (20 mg/kg i.p.) and then received DOXO (10 mg/kg i.p.) every other day and were sacrificed 7 days after the treatment.

Mice that received saline were used as control group.

At the end of each experimental time, heart samples were collected and prepared for molecular biological analyses.

### 4.4. Echocardiogram

Ejection Fraction (EF), Fractional Shortening (FS), Left Ventricular End-Diastolic-Diameter (LVEDD), and Left Ventricular End-Systolic Diameter (LVESD) were evaluated echocardiographically in mice at baseline and before sacrifice. Mice were anesthetized (1–1.5% of isoflurane in oxygen) and the heart rate stabilized to 400–500 beats per minute. Echocardiography was performed by means of VEVO (VisualSonic, Toronto, ON, Canada) instrument and analyzed by means of the VEVO analysis software (Toronto, ON, Canada).

### 4.5. Protein Extraction and Western Blot Analysis

Total proteins were extracted by homogenization of hearts with a dounce potter in lysis buffer containing TRIS-HCl (50 mM), NaCl (500 mM), protease inhibitors, PMSF (0.25 μM), NaF (50 mM), Na_3_VO_4_ (0.2 mM). Protein concentration of lysates was determined using a protein assay kit (BIO-RAD, Milan, Italy). Fifty micrograms of protein were applied to each lane of a 10% SDS polyacrylamide gel, subjected to electrophoresis, and then transferred to nitrocellulose paper (GE Healthcare, Björkgatan, Sweden). Membrane strips were blocked for 1h at room temperature in blocking solution (NaCl/TRIS, 0.1% Tween 20 (*v*/*v*), 5% powdered milk (*w*/*v*) and then blotted with primary antibody anti-Cx43 (Sigma, 1:8000), anti-pCx43 phosphorylate on Ser368 (Santa Cruz, 1:250), anti-sarco/endoplasmic reticulum Ca2^+^-ATP_ase_ (SERCAII; Santa Cruz, 1:250), anti-phospholamban (PLB; Santa Cruz, 1:250), or anti-glyceraldehyde 3-phosphate dehydrogenase (GAPDH; Santa Cruz, 1:1000, used as loading control) overnight at 4 °C. After three washes with PBS 0.1% Tween, the membranes were incubated for 1 h at room temperature with secondary antibody anti-rabbit, anti-mouse, or anti-goat (each diluted 1:4000). After three washes with PBS 0.1% Tween immunoreactive proteins were revealed by an enhanced chemiluminescence reagents (ECL) in LAS 4000 (GE Healthcare).

### 4.6. Mitochondrial Protein Extraction and Western Blot Analysis for Mitochondrial Cx43 and pCx43 

Mitochondrial proteins were isolated from mice heart by means of a dounce potter in a lysis buffer solution holding EGTA (1 mM), K^+^ Hepes pH 7.5 (20 mM), MgCl_2_ (1.5 mM), sucrose (250 mM), EDTA (0.1 mM), KCl (10 mM), protease inhibitors, PMSF (100 μM), Na_3_VO_4_ (0.2 mM), NaF (50 mM), DTT (1 mM), digitonin 0.025%. The amount of protein contained in the lysates was evaluated by means of protein assay kit (BIO-RAD, Milan, Italy). Cx43 and pCx43 expression was evaluated by Western blot analysis, as previously described. Primary antibody anti-TOM20 (Santa Cruz, 1:250) was used as loading control. The purity of mitochondrial protein extraction was performed by means of Western blot analysis by evaluation of the presence of proteins expressed only in the mitochondria (ox-Phos Complex II, Abcam, 1:7000) and the absence of proteins expressed in other cellular compartments (Na^+^/K^+^ ATP_ase_, Abcam, 1:3000) [45].

### 4.7. Primary Cardiomyocytes Isolation and Measurement of Intracellular Ca^2+^ Signaling

Primary cardimyocytes were isolated from heart of mice treated as previously described and from heart of control mice to evaluate intracellular Ca^2+^ concentrations. After a wash with Hank’s balanced salt solution (HBSS) 0.1 mM Ca^2+^ containing NaCl (140 mM), KCl (5.4 mM), KH_2_PO_4_ (0.44 mM), Na_2_H PO_4_ (0.42 mM), NaHCO_3_ (4.17 mM), CaNa-EDTA (26 mM), CaCl_2_·H_2_O (0.10 mM), HEPES (5.0 mM) and dextrose (5.5 mM), heart were cutted in 1–2 mm sections and incubated in HBSS with the addition of albumin (10 mg/mL), tryspin inhibitor (1 mg/mL), taurine (5 mM), dithiothreitol (0.4 mg/mL), collagenase II (0.6 mg/mL), and papain (0.6 mg/mL). Following an incubation period of 75 min at 37 °C, the suspension was filterd (0.70 µ filter) to remove the cardiac fragments and centrifuged to pick the cardiomyocytes. Primary cardiomyocytes (3 × 10^4^ cells/mL) were re-suspended in 1 ml of HBSS containing the ratiometric fluorescent indicator dye FURA2-AM (5 µM) and incubated for 45 min at 37 °C. Then, cells were washed with HBSS to remove the excess of FURA2-AM and incubated for further 15 min in Ca^2+^-free HBSS/0.5 mM EGTA buffer to allow hydrolysis of FURA2-AM into its active-dye form, FURA2. Intracellular Ca^2+^ levels were then analyzed spectrofluorimetrically (Perkin-Elmer LS-50). Excitation wavelenghts for FURA2 were 340 and 380 nm and the emission wavelenght was 515 nm. Data are reported as delta increase of fluorescence ratio of 340 nm/380 nm induced by stimulus (ionomycin, 1 µM; thapsigargin, 1 µM; carbonyl cyanide p-trifluoromethoxy-pyhenylhydrazone, FCCP, 0.05 µM)—basal fluorescence ratio of 340 nm/380 nm, and is referred as Ca^2+^ signal. 

### 4.8. Immunohistochemical Analysis

Frozen cardiac tissue (7 µm), fixed in 4% paraformaldehyde in PBS, were permeabilized with Triton X (0.1% *v*/*v* in PBS), blocked with Bovine Serum Albumin (5% *v*/*v* in PBS) and then incubated with mouse anti-Cx43 and rabbit anti-TOM20 for 2 h at room temperature. After three washing steps with PBS, the sections were incubated with secondary antibodies (FITC-conjugated anti mouse IgG and Texas red-conjugated anti rabbit IgG) for 1 h at room temperature in the dark. DAPI was used to detect the nuclei. Afterwards the slides were mounted and were observed by using Laser Confocal Microscope (Leica TCS SP5, Wetzlar, Germany).

### 4.9. Statistical Analysis

Data are expressed as mean ± standard error mean (SEM) of at least three independent experiments. Statistical analysis were made with Student’s *t* test and *p* < 0.05 was considered significant.

## Figures and Tables

**Table 1:** Effect of DOXO (10 mg/kg; i.p.) or DZX (20 mg/kg; i.p.) or combined DZX+DOXO treatment on Ejection Fraction (% EF), Fraction Shortening (% FS), Left Ventricular End-Diastolic Diameter (LVEDD) and on Left Ventricular End-Systolic Diameter (LVESD), after a single administration (1th group), two administrations (2nd group), or three administrations (3rd group). Results were expressed as mean ± S.E.M. from 4 mice/group. Data were analyzed by Student’s *t*-test. * *p* < 0.05, and ** *p* < 0.005 vs. control; and # *p* < 0.05 DZX+DOXO vs. DOXO.

**24 h**		**Control**	**DOXO**	**DZX**	**DZX+DOXO**
	LVEDD	3.97 ± 0.11	4.09 ± 0.1	3.8 ± 0.010	3.725 ± 0.145
	LVESD	2.62 ± 0.17	3.00 ± 0.06 *	2.8 ± 0.40	2.67 ± 0.146
	%EF	62.17 ± 4.1	52.7 ± 1.38 **	53.0 ± 5.0	54.25 ± 1.8 #
	%FS	30.41 ± 0.72	26.6 ± 0.92 *	27.0 ± 3.000	27.5 ± 0.936 #
**3 days**		**Control**	**DOXO**	**DZX**	**DZX+DOXO**
	LVEDD	3.940 ± 0.050	3.96 ± 0.06	3.895 ± 0.005	3.792 ± 0.086
	LVESD	2.780 ± 0.054	2.90 ± 0.056 *	2.70 ± 0.400	2.823 ± 0.107
	%EF	57.20 ± 1.25	53.73 ± 1.61 *	70.0 ± 1.000	50.500 ± 2.217
	%FS	30.410 ± 0.850	27.43 ± 1.02 *	39.5 ± 0.500	25.000 ± 0.354 #
**7 days**		**Control**	**DOXO**	**DZX**	**DZX+DOXO**
	LVEDD	3.860 ± 0.040	3.96 ± 0.055 *	3.515 ± 0.055	3.710 ± 0.079
	LVESD	2.730 ± 0.150	2.9 ± 0.14	2.350 ± 0.350	2.553 ± 0.135
	%EF	59.000 ± 4.170	50.49 ± 4.79 **	63.0 ± 2.000	57.00 ± 2.606 #
	%FS	31.760 ± 1.680	25.39 ± 3.02 *	32.00 ± 2.000	29.000 ± 1.517 #
